# Magnetic resonance imaging in sudden deafness

**DOI:** 10.1016/S1808-8694(15)31193-9

**Published:** 2015-10-20

**Authors:** Hugo Valter Lisboa Ramos, Flávia Alencar Barros, Hélio Yamashita, Norma de Oliveira Penido, Ana Cláudia Valério de Souza, Wellington Yugo Yamaoka

**Affiliations:** 1 Otorhinolaryngologist, Post-graduation studies under course.; 2 Speech therapist and audiologist, Department of Otorhinolaryngology and Head and Neck Surgery, Federal University of Sao Paulo/ Escola Paulista de Medicina.; 3 Ph.D. in Medicine, Professor, Department of Imaging Diagnosis, Federal University of Sao Paulo/ Escola Paulista de Medicina.; 4 Ph.D. in Medicine, Professor, Department of Otorhinolaryngology and Head and Neck Surgery, Federal University of Sao Paulo/ Escola Paulista de Medicina.; 5 Post-graduation studies in Otorhinolaryngology and Head and Neck Surgery, Federal University of Sao Paulo/ Escola Paulista de Medicina.; 6 Post-graduation studies in Otorhinolaryngology and Head and Neck Surgery, Federal University of Sao Paulo/ Escola Paulista de Medicina.

**Keywords:** sudden deafness, magnetic resonance imaging, Ménière’s disease, vestibular schwannoma

## Abstract

The etiology of sudden deafness can remain undetermined despite extensive investigation. This study addresses the value of magnetic resonance imaging in the analysis of sudden deafness patients. **Study design:** transversal cohort. **Material and Method**: In a prospective study, 49 patients attended at otolaryngology emergency room of Federal University of Sao Paulo - Escola Paulista de Medicina, from April 2001 to May 2003, were submitted to magnetic resonance imaging. **Results**: Magnetic Resonance abnormalities were seen in 23 (46.9%) patients and revealed two tumors suggestive of meningioma, three vestibular schwannomas, thirteen microangiopathic changes of the brain and five (21.7%) pathological conditions of the labyrinth. **Conclusion**: Sudden deafness should be approached as a symptom common to different diseases. The presence of cerebellopontine angle tumors in 10.2% of our cases, among other treatable causes, justifies the recommendation of gadolinium-enhanced magnetic resonance use, not only to study the auditory peripheral pathway, but to study the whole auditory pathway including the brain.

## INTRODUCTION

Sudden deafness (SD) is a symptom that brings a number of challenges to the clinician faced by it. The etiology of sensorineural sudden loss can not always be defined even making use of all diagnostic tools as we have today. There are many complementary examples used to define the cause, such as audiological, biochemical, otoneurological or imaging tests.

Magnetic resonance imaging (MRI) has been the preferred imaging exam to study in details the inner ear structures, inner acoustic canal and cerebellopontine angle. However, little has been said about the value of MRI in studying the central nervous system (CNS) in patients with SD. In 495 cases of sensorineural loss studied with MRI, Wu and Thuomas^1^ identified 22 cases of intra-axial lesions calling our attention to the etiology of central deafness. Moreover, the possibility of audiovestibular pathway lesions as the central cause of sudden deafness has been considered by other authors ^2^.

In this study, we will show you the study of MRI in patients with SD. We will assess frequency and characteristics of the detected affections.

## MATERIAL AND METHOD

This was a prospective study developed by the Department of Otorhinolaryngology and Human Communication Disorders and the Department of Imaging Diagnosis, Escola Paulista de Medicina, Federal University of Sao Paulo, between April 2001 and May 2003.

Patients with complaints of sudden hearing loss or loss for 72 hours underwent audiometry and immittanciometry. We considered the diagnosis of sudden deafness as a unilateral sensorineural loss, equal or greater than 30 dB in at least three consecutive audiometric frequencies. Subjects that presented auditory losses with other characteristics were excluded from the study. We included in the study patients of both genders who had no age restrictions.

The studied patients were submitted to temporal bone and brain MRI performed with device Philips Gyroscan NT 1.5 tesla. Images were obtained in a sequence concentrated in T1 Turbo Spin Eco (TSE) at axial and coronal plans, with and without contrast, in 2.5mm thick sections. We also collected T2 images at coronal plan in TSE, with 2.5mm thick sections, and in 3D using 0.7mm reconstructions. We complemented the exam using brain axial sections in a concentrated sequence in “Fluid Attenuation Inversion Recovery” (FLAIR) to assess the brain. We considered as peripheral affections all those limited to the inner ear, and central ones as those located in the internal acoustic canal (IAC) or the central nervous system (CNS).

Results were descriptively analyzed.

## RESULTS

Sixty-one patients were included in the study. Out of the total, 12 dropped out or were not submitted to MRI for other reasons. Only 5 of the 49 patients managed to undergo MRI before clinical treatment, which was performed with prednisone and pentoxifylline. Patients’ ages ranged from 15 to 91 years with mean age of 45.4 years, 23 (46.9%) were male and 26 (53.1%) were female. There were 23 patients (46.9%) affected on the left side and 26 (53.1%), on the right side. As to race, 34 (69.3%) patients were Caucasian, 10 (20.4%) were native Brazilian, 2 (4.1%) were African-descendents and 3 (6.1%) were Asian-descendents.

Out of the total of 49 studied patients, 23 (46.9%) presented MRI abnormalities. Among the affected MRI, the mean age was 55.1 years and 11 (47.8%) were male and 12 (52.2%) were female patients. We found 24 abnormalities as a whole, because one patient presented two findings.

**Figure 1:**
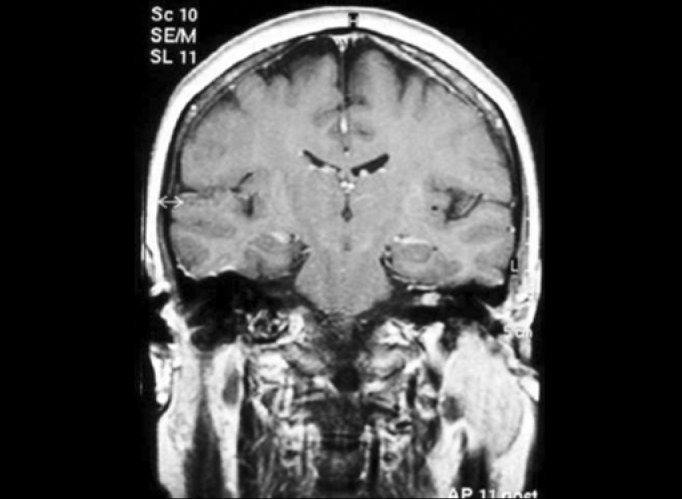
Intra-canalicular Schwannoma - MR (T1 sequence with gadolinium).

**Figure 2:**
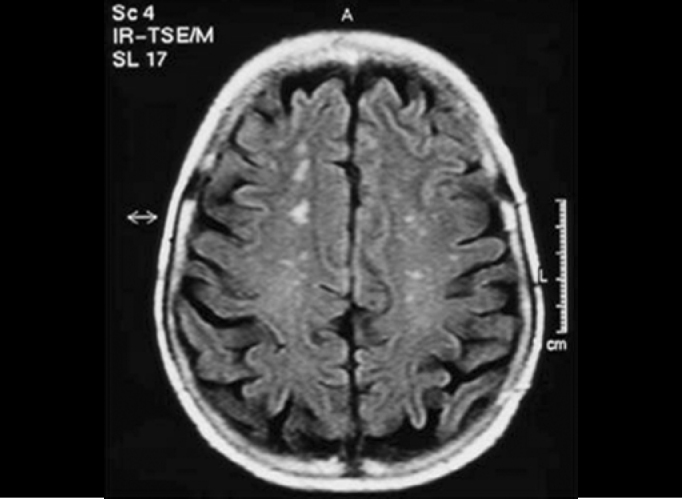
Hyperintense subcortical lesions - MR (FLAIR).

We found two tumors suggestive of meningioma in the para-sellar region, which were extended inferiorly and three tumors with characteristics of 8th cranial nerve schwannoma, and two of them were intra-canalicular (Figure 1). Another patient presented dilation of the 4th cerebral ventricle. However, the most frequent central finding was presence of occasional subcortical and periventricular lesions, which were hyperintense in FLAIR, detected in 13 patients (Figure 2).

**Figure 3:**
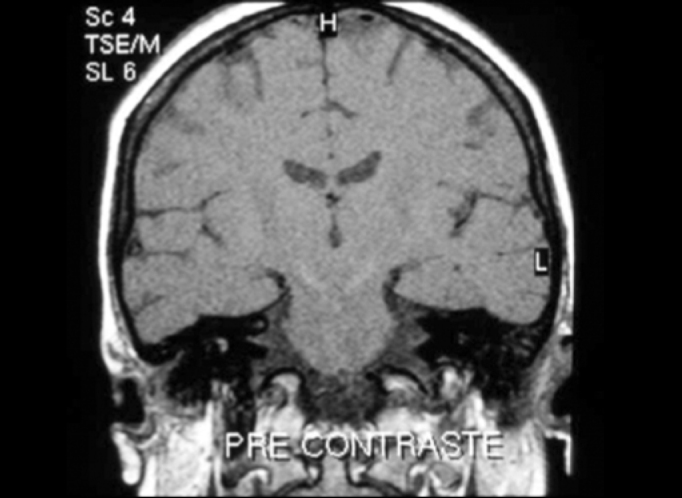
Vestibulocochlear enhance to gadolinium - MR (T1 sequence) - pre-gadolinium.

**Figure 4:**
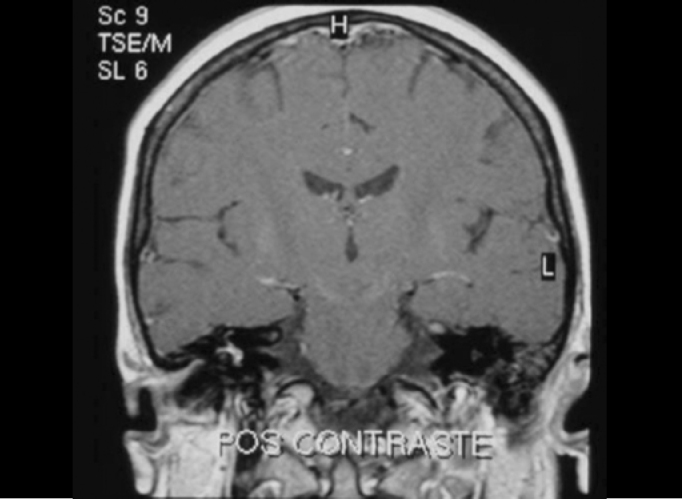
Vestibulocochlear enhance to gadolinium - MR (T1 sequence) - post-gadolinium.

**Figure 5:**
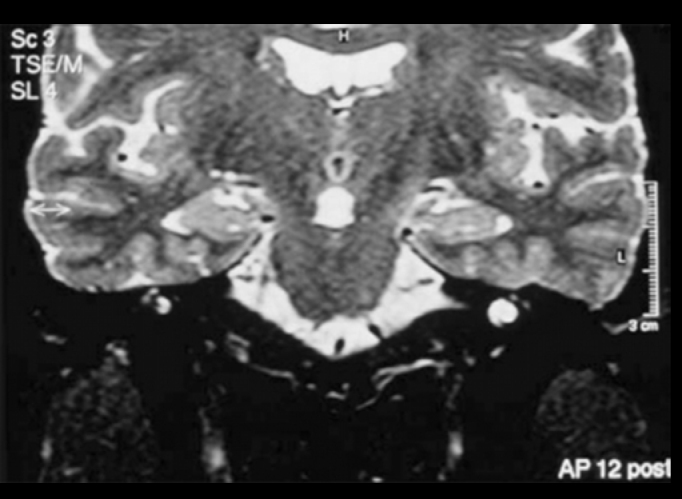
Hyperintense vestibulocochlear signal - MR (T2 sequence).

Five (21.7%) patients presented peripheral affections and they were all homolateral to deafness. Two types of affections were found: vestibulocochlear enhance to gadolinium use observed in T1 sequence (Figures 3 and 4) in two patients and vestibulocochlear hypersignal observed in T2 sequence, with or without use of contrast (Figure 5) in 3 patients. In addition to peripheral lesions, one of these patients presented hyperintense subcortical lesions, already described, and in one patient we identified cervical vertebral C2 nervous root schwannoma, a finding which was not considered in the calculation.

## DISCUSSION

Despite the wide spectrum of exams used in the study of audio-vestibular affections, the diagnosis of the possible causes of sudden deafness remains a challenge. The introduction of MRI in the diagnosis of sudden deafness has contributed to the detection of lesions that had never been diagnosed before. MRI is currently considered the exam of choice in radiological investigation of the inner ear, vestibular-cochlear nerve and central nervous system. Schick et al.^2^ identified abnormalities using MRI in 34.5% of 354 exams of patients with SD, non-pulsatile tinnitus and vestibular symptoms, meaning that it is useful in the investigation of audio-vestibular disorders.

The initial symptom in about 10% of the patients with vestibular schwannoma is of SD 3, 4. However, low prevalence of this tumor among patients with SD and the high cost of MRI are factors that have discouraged its use. Inoue et al.^3^ showed that in 24 studied patients with schwannoma, 20.8% recovered hearing completely with clinical treatment. This fact describes the importance of MRI in cases in which there is recovery of hearing, given that it does not imply a simple disorder of the hearing system. In our study, one patient with intra-canalicular schwannoma presented completely recovery of deafness with clinical treatment.

Wu and Thuomas^1^ reinforced that we should give attention to cerebellopontine angle and inner acoustic canal rather than the cochlear and intra-axial auditory pathway. In a study with 495 MRIs, they showed findings of intracranial lesions in 42.6% of the cases, and it was intra-axial in 23.1% of them. They also pointed out the fact that the lesions they found were responsible for SD in 19.2% of the patients, which has definitely clarified the diagnosis.

Mark et al.^5^ studied 12 patients with SD and discussed that the finding of labyrinth enhance in the affected side to the use of gadolinium was indicative of peripheral disease and allowed the definition of anatomical diagnosis of the affection.

In our study, 46.9% of the patients showed MRI affections. In 20.83% of the cases, there were inner ear affections and in 79.17% of the patients, findings were not central. Even though peripheral findings had occurred as less frequent, the side of affection was the same as the side of deafness in 5 patients. We observed two types of peripheral affections: vestibulocochlear enhance to the use of gadolinium, observed in T1 coronal sections and vestibulocochlear hypersignal, when compared to the contralateral side, seen in T2. Traditionally, enhance to use of contrast has been radiologically interpreted as a signal of affection in the chemical composition of inner ear liquids, which was indicative of inflammatory lesions of the inner ear, of viral or immunomediated cause ^6^. Low incidence of these peripheral affections found in our study has certainly not been a perfect representation of the reality, probably owing to the previous treatment with prednisone in 45 to 49 studied patients.

Conversely, vestibulocochlear hypersignal observed in T2 is a finding described by Kano et al.^6^ in patients with SD and suggested the presence of higher amount of liquid in the labyrinth in comparison with the contralateral ear. By analyzing in details the images, we observed that these patients have also presented a distance between the vertical portion of the posterior semicircular canal and the posterior fossa of the skull similar to that found in patients with Ménière disease, described by many authors 7, 8. We found mean of 2.7mm in the 3 reported cases. Mateijsen et al.^9^, in a study with 86 patients compared to 62 controls, observed that the mean distance in patients with Ménière disease was 2.9mm and in the controls it was 3.8mm (p<0.001), and that unilateral cases had smaller values. We suspected that vestibulocochlear hypersignal also has a radiological signal for endolymphatic hydrops, a signal that would surge in cases of sudden deafness, evidencing severe situations of hydrops decompensation of the labyrinth. Either way, these findings indicate a pathophysiological substrate for deafness.

Out of 49 studied patients, there was one confirmed case of viral labyrinthitis. One patient aged 15 years presented deafness one week after the onset of symptoms of mumps and progressed without improvement of hearing thresholds. He also presented orchitis, which made him infertile. This patient showed vestibulocochlear enhance to contrast seen in T1, ipsilateral to deafness, indicating the cochlear affection by the virus.

Hyperintense subcortical and periventricular lesions in FLAIR sequence were the most frequent central lesions (68.4% of central lesions). This type of affection was found in 22% of the 354 patients studied by Schick et al.^2^ and it is considered as a signal of microangiopathic cerebral lesions. Vascular affections have been one of the most controversial etiologies of SD and some authors do not believe they should be held responsible for SD. Experimental studies have demonstrated that embolization of cochlear vessels produce ossification and extensive cochlear fibrosis, findings which are not common in humans patients with SD 10, 11. Vasama and Linthicum^12^ studied, through histopathological examination, 12 temporal bones of patients with SD, comparing them to normal patients and subjects with presbycusis. The authors stated that vital etiology seemed to be one of the most common causes of SD, and in addition they exclude a possible vascular cause that could explain their findings. However, 4 patients in the study, or one third of the sample, presented different histopathological findings, so other likely etiologies, in addition to viral, were suggested by the authors. Conversely, Yoon et al.^13^ described different histopathological findings in two patients with suspicion of vascular cause. The authors discussed that some differences in findings of inner ear are found in SD of vascular origin and can be owed to variations in severity of vascular insults or even to different pathogenic mechanisms.

Differently from the studies above reported, some cases of vascular affections have been described. Watanabe et al.^14^ described two cases of SD caused by vertebral-basilar artery affection: the first patient presented SD after super-selective embolization of vertebral artery and the second after accidental ligation of one of the cerebral arteries. Ohinata et al.^15^ showed a study with 51 patients comparing them with 70 controls and patients with SD presented increased blood viscosity, which was positively correlated with level of hearing loss. The authors concluded that normalization of blood viscosity is essential in treating SD.

In our study, patients with hyperintense subcortical and periventricular lesions had higher age mean (65.2 years) and even though there was no vascular affection, we believe that these affections detected in the MRI indicated vascular etiopathogenesis for deafness.

Five (21.7%) patients presented intracranial tumor lesions and in 5 cases the tumor could be held responsible for sudden deafness. One patient had already been submitted to partial exeresis of meningioma for 10 years, but tumor growth towards cerebellopontine angle was responsible for sudden deafness after one decade of follow-up. In other two patients, there was complete recovery of deafness with clinical treatment despite the presence of tumor. This small rate of tumor lesion found in our study should not discourage the use of MRI. Its importance in the identification of intra-canalicular tumors has already been defined and for this reason MRI has a role in differential diagnosis of tumor lesions in SD.

Thus, we can see that by studying the labyrinth, 8th nerve and CNS, in different areas, we can defined the etiological diagnosis of SD and MRI is still the best complementary exam currently available.

## CONCLUSION

SD should be approached as a common symptom of different diseases and for this reason, the search for the etiology of the hearing loss is indispensable in all cases of acute sensorineural hearing loss. The presence of cerebellopontine angle tumors in 10.2% of our cases, among other treatable causes, justifies the use of contrast both in the study of the peripheral hearing system and in the study of the central auditory pathways, including the brain.
